# National Dental Association

**Published:** 1900-01

**Authors:** 


### Reports of Society Meetings.

### NATIONAL DENTAL ASSOCIATION.

(Continued from Vol. XX., page 795).

### *Second Day.—Evening Session.*

THE Association was called to order at 7.30 P.M., Vice-President S. H. Guilford taking the chair. The report of the Committee on the President's Address was read by the chairman of the committee, Dr. James McManus.

On motion of Dr. Crouse, the proposed amendments to the constitution were referred to the committee on revision and the report of the committee was received.

After the transaction of some minor routine business the hall was darkened for the exhibition of stereopticon views, and Dr. C. Edmund Kells, New Orleans, read an illustrated paper entitled "Röntgen Rays."

Dr. Kells, in his paper, reviewed the history of this marvellous discovery, beginning with a sketch of the study by Dr. William Crookes of the properties of matter in high vacua, and the construction of the "Crookes" tube. For seventeen years the studies of scientists all over the world were devoted to the effects produced within the latter by the cathode ray, Wilhelm Röntgen, of Würzburg, being the first to discover the more important effects produced beyond the confines of the glass walls of the Crookes tube. The history of this great discovery was given briefly by Dr. Kells, and illustrated by pictures thrown upon the screen of the apparatus for exciting the tube, and of the means for utilizing the "X-ray" as it is produced. Dr. Kells also described the means devised by himself to prevent the puncture of the tubes when used upon the Tesla coil. The paper concludes with an interesting *résumé* of the uses of the Röntgen ray in dentistry and the requirements for obtaining the best results in the least time. By Dr. Kells's methods the time for producing a "radiograph" or "skiagraph" of unerupted teeth, etc., runs from twenty to forty seconds in the thinner bones to as high as one hundred and twenty seconds for third molars in heavy jaws, several pictures being obtained at the same time, if desired, by using several celluloid films superimposed one upon the other.

Other papers read at this session were "A Study of Harelip and Cleft Palate" (illustrated), by Dr. Thomas Fillebrown. "Some New Points in the Anatomy of the Face and Jaws" (illustrated), by Dr. M. H. Cryer. (These papers require the illustrations.)

Dr. Grevers, of Amsterdam, read a paper on "Dental Nomenclature." This paper was illustrated by photographs projected upon the screen showing the different occlusal conditions, normal and abnormal, for which Dr. Grevers proposes a nomenclature based upon the Greek root with various prefixes, as, *en*-armosis, normal bite; *eph*-armosis, projecting mandibulæ; *pros*-armosis, edge to edge bite; *oph*-armosis, open bite; *dieh*-armosis, cross bite; *odorith*-armosis, occlusion of the teeth, etc.

### *Third Day.—Morning Session.*

The Association was called to order at 9.30.

Dr. H. A. Smith, Chairman of the Committee on Code of Ethics, presented a code based upon that adopted by the American Dental Association in 1866, which, after some revision, offered by Drs. Harlan, Patterson, and Taft, was unanimously adopted.

Dr. J. Y. Crawford offered the following resolution, which, after discussion by Drs. Jarvis, Truman, and Gordon White, was adopted:

"WHEREAS, The National Dental Association deems it a duty to express its condemnation of the continued forcing of politics with the formation of State Examining Boards; be it

*Resolved*, That while such bodies are of service to the public when composed of strictly competent men, they are a decided evil, and a disgrace to dentistry, when self-seeking and unprofessional individuals are placed upon such boards solely through political influence."

Dr. Stainton, chairman of the committee appointed to examine the conduct of the affairs of the Dental Protective Association, reported that the committee had examined the books and vouchers of the Association and found everything correct. It was to be regretted, however, that but eight hundred members had paid the second assessments permitted by the by-laws of the Association.

Dr. W. W. Walker desired that Dr. Crouse would be asked to explain the meaning of an article in the New York *Herald* in regard to a law-suit claimed to have been just won by the Sheffield Tooth Company.

Dr. Crouse stated that the attorney of the Association would be in attendance on Friday morning, when a meeting of the Dental Protective Association would be held. All members of the profession were invited to be present, as matters of the greatest importance to every dentist would be presented.

Dr. Wedelstaedt, of St. Paul, read a paper entitled "Cements."

He said that cements having been in use for the past thirty years, it seemed strange that it had not been recognized that the majority of those in use are very readily penetrated by moisture. If each man, the next time he uses cement, will mix a little more than is needed and place the residue in a bottle of ink for from twenty-four to forty-eight hours, he can readily ascertain the extent of penetration of moisture. If he finds one that is not penetrated, that is a good one to stick to. If the ink can penetrate a pellet of the cement, saliva can penetrate it, and where saliva can penetrate, micro-organisms can penetrate also.

Dr. Wedelstaedt then produced a number of glass tubes containing an aniline solution, in which, on the 12th of July, masses of mixes of different cements had been placed.

Those in one tube had been covered with sandarac varnish; another in hot melted paraffin; the third had no preparation.

Dr. C. N. Johnson was requested to remove the cement cubes and report the result.

1. Those without varnish or paraffin showed considerable penetration by the aniline.

2. With sandarac there was some penetration.

3. With hot paraffin the penetration was also quite considerable. The "fillings" or masses of cement were each seven millimetres in diameter by twenty-two millimetres long.

After the examination Dr. Wedelstaedt further said that penetration was but one of the evils to be overcome; there was also contraction and expansion of the mass, some of his experimental fillings showing an expansion of two millimetres beyond the cavity, while in others there was marked contraction running completely around the filling. Various experiments were made, as by mixing the powder of a cement that leaks with the liquid of one that expands, and *vice versa.* A microscopic study of the powder of different cements gave some surprising results, bits of wood, hair, cotton, etc., having been found.

Age also has a remarkable effect upon cement fillings, as tested by the dynamometer, the resistance increasing very rapidly with age. In one case, in which a cement carried but thirty or thirty-five pounds at the end of twenty-four hours, more of the same mix carried four hundred pounds after ninety-six hours, the practical lesson being not to place metal fillings on freshly placed cement.

When we make a demand for cements that will neither shrink nor expand, and which moisture will not penetrate, we will get them, and the better it will be for our patients.

From the examination of the samples presented, Dr. Johnson said that not one of the cements experimented with was fit to use as filling-material for teeth.

Dr. W. V. B. Ames, Chicago, read a paper entitled "Some Phases of the Cement Question."

He said that in the consideration of the physical properties of filling-materials the cements should have a share of attention. The paper dealt with the questions of the crystallization of cement liquids, of coarse *versus* fine powders, of shrinkage and expansion, and of the arsenical contamination of cement powders. The crystallization of the liquid has been the bugbear of cement manufacturers. Crystals separate from the fluid, either held in suspension, settling at the bottom of the bottle, or adhering to its sides. As these crystals were originally part of the solution, evenly distributed throughout, their separation and loss must impair the virtues of the original formula. They may, however, usually be liquefied by heat, or, if everywhere distributed through the liquid, may be rubbed down and liquefied in the mix of the cement. As to the degree of fineness of the powder in dynamometer tests there is an increase of edge-strength up to a point somewhat short of impalpability. We may expect greater strength of contour when a slight grit is discernible, but it is reasonable to suppose that the granular state may detract from the ability of the mass to withstand long-continued attrition. Powder that is too coarse for inlay-setting may be suitable for contour.

On the question of shrinkage and expansion he said that when the phosphoric acid has been modified by alkaline phosphates only, the basic phosphate which is formed is of a friable nature, and exerts no special force in drawing together the granules, making evident shrinkage at the periphery. But when the acid has been modified by non-alkaline phosphates, the basic phosphate formed agglutinates the zinc oxide granules, drawing them towards the centre of the composition, giving a tendency to a diminution of volume towards crystallization, depending on a lack of water of crystallization which, if present, would give too rapid setting, but which, if added to the crystallizing mass, will be taken up and give the difference between shrinkage and expansion. When the mass is allowed to harden in a tube in the dry state, wholly unlike conditions present to those existing when the same cement is used in the mouth. The practical lesson from this is that cement fillings are not benefited by being kept dry for an indefinite period, that there is a disadvantage rather than an advantage in long-continued protection from the saliva, in the use of the cements in which the phosphoric acid is modified by non-alkaline phosphates.

The arsenical contamination of cement powders is easily demonstrated, but the zinc-arsenic compound is inert and wholly devoid of poisonous properties *per se*, and is not broken up to the extent of forming potent arsenous acid except under rare conditions, so that the infinitesimal arsenical contamination may be ignored.

In repeated tests it has been proved that the death of a tooth-pulp will not be caused even by the long-continued proximity of a liberal portion of this zinc-arsenite. Sensitiveness of dentine is not decreased by its application, and the pulp subsequently presents all the appearances of normal vitality.

In the discussion of the subject, Dr. G. V. Black said that it was greatly to be desired that the physical properties of the cements should be investigated by competent persons, for, although it is being looked into, it has not yet been shown that we have a reliable cement to-day, one that will do what cement fillings are expected to do. They are utterly unreliable even for covering in arsenical applications. Unless each batch is tested we do not know whether it is going to shrink or expand. From observations made in his own laboratory he has found that in a well-prepared cavity there was sufficient shrinkage in an hour or so to let enough arsenic out on to the gum to cause arsenical poisoning, a shrinkage that could be seen with an ordinary magnifying-glass. We should therefore use the utmost caution until assured by experience of the quality of our cements.

*Dr. Patterson* said he did not see how it could have been possible for Dr. Wedelstaedt to make a perfect mix of cement in such large masses. The cylinders are chalky and crumbling.

*Dr. Wedelstaedt* said that only one of the "fillings" presented was made from a mix, and that the mixing was thoroughly done.

*Dr. Crouse* hoped that more men would take up this work, so that we would eventually have a cement that would not shrink, that would not be porous, and from under which arsenic could not escape. As yet we know but very little about the properties of the cements, although we use them daily and rely upon them more or less.

In closing the discussion of his paper, Dr. Wedelstaedt described more exactly the methods used in making his test fillings, and said that it was only through such experimental work that one could hope to obtain an improved cement.

*Dr. Ames* said that from his own work in this direction he had reason to believe that the tendency to shrinkage would yet be overcome, and that we would eventually have a cement that would be impervious to moisture.

*Dr. G. V. Black*, Chicago, next read a paper entitled "Susceptibility and Immunity to Dental Caries."

These questions have not had the amount of consideration by dentists that their importance demands. Caries is and has been so common that it has come to be considered that practically all are so afflicted.

And yet there are a sufficient number of exceptions for all who have been long in practice to have known some persons who have been wholly immune during a long life. This has been in the past attributed to the superior quality of the teeth; but it has been proved that this is erroneous. Caries is not dependent upon the teeth, for all human teeth are nearly the same in their calcification, though in their physical structure there are wide differences,—developmental grooves are imperfectly closed, leaving fissures, pits, and openings; the dentine may have interglobular spaces; granular areas in which there is much of physical imperfection. But these imperfections are in no proper sense a *cause* of caries of the teeth. They are proportionately as frequent in persons immune to caries as in persons whose teeth decay. These imperfections at most only give opportunity for the action of the causes of decay when the cause is present and active. It is an error to look to the teeth themselves for the answer to the question *why* some teeth decay and others do not. In caries the teeth are acted upon; they do not themselves act in the premises. The agents acting to produce decay are outside of the teeth, and must be found in their environment,—*why* the cause which is active in some and not in others has not been reduced to demonstration. It is certain that caries has its beginning only when the conditions of the oral secretions are such that the micro-organisms causing decay form gelatinous plaques by which they are glued to the surface of the teeth. This seems necessary to the starting of the process of decay. So long as the micro-organisms have no protection from the dissipation of the acids they form they cannot produce caries. If the saliva was even sufficiently acid to act upon the teeth they would decay all over instead of at selected points of beginning. The organisms grow in every mouth, but the formation of the gelatinous plaques does not occur in every mouth, and this seems to depend upon something in the saliva, the nature of which is yet unknown. The gelatinous plaque of the caries-fungus is a thin, transparent film, revealed only by careful research, and not to be confounded with the thick mass of *materies alba* so frequently found upon the teeth; nor is it the whitish, gummy material known as *sordes*. Research for the factors which predispose to caries should be directed not to the teeth themselves, which are the most unchanging of the tissues of the human body, but to the surroundings of the teeth,—the oral fluids and the bodily conditions which give character to the secretions, which are affected by the slightest causes. With our present knowledge it may be stated that the susceptibility to caries of the teeth is influenced by heredity, by age, and by fluctuations of bodily condition. The hereditary predisposition to caries is of first importance, as with children living under conditions similar to those of the parents in their childhood the susceptibility to caries will be very similar in the majority of cases, even to the particular teeth and the localities liable to be first attacked. Caries in its most practical aspects is a disease of youth. The hereditary predisposition disappears with the coming of adult age. The beginning of nearly all cavities occur early in life, and the effect of early radical treatment by filling is that the person becomes immune at the time of life at which caries would be making its worst ravages if the filling had not been done. This immunity relates to the beginning of caries rather than to the progress of cavities that have begun. This is partly due to better care, the better care being due to the better conditions which rendered the better care possible, the teeth being placed in condition for full and natural use in the mastication of food and the natural abrasive action of the excursions of food with its effect in natural cleansing. This is the most important element in bringing about an early abatement of susceptibility to caries. Reacting in a general way, a better condition of the secretions is produced. It is for children, therefore, that the most perfect operations are demanded in order to maintain the full, free, and efficient use of the teeth. A failure of vigorous mastication seriously retards the coming of immunity. Clinically, the results support the practice of making permanent fillings for children, although this has been severely criticised. It is true that the pulp occupies larger space, but this simply demands corresponding care and judgment. Judicious management is required, but if proper means are employed the endurance of the child will be sufficient; but the courage of a child should never be broken down, and the nervous system should be looked to with the greatest care. When from any cause whatever permanent operations cannot be made, the case must be tided along with palliative treatment until better conditions obtain.

Fluctuations between susceptibility and immunity occur, not dependent upon changes in the tissues of the teeth, but to changes in the environment of the teeth, in the secretions, and, possibly, in the cellular elements of the general body. These changes in the character of the secretions are often coincident with what seems to be the best of health. That they are frequently coincident with ill health of one kind or another seems to be merely a coincidence, and it does not appear that any especial form of ill health particularly predisposes the teeth to caries. Local immunity is due not to difference in the tissues of the teeth in the favored localities, but rather to environment, certain localities favoring the building and protection of the microbic plaques.

Without careful consideration of the question of environment and comparison with what occurs in conditions of susceptibility, clinical experience will lead to conclusions very wide from the facts.

*Dr. J. T. Crawford* (Chairman of the Section).—From the moral and the philosophic stand-point the papers of Dr. Black and Dr. Johnson are so nearly alike that I will ask that Dr. Johnson be permitted to read his paper now, and that the two be discussed conjointly.

*Dr. C. N. Johnson* accordingly read his paper, entitled "Management of Children's Teeth."

In the management of the deciduous teeth the object is usually to do merely palliative work, keeping the patient comfortable for a few years until the permanent teeth have taken their place.

With the permanent teeth, even those which appear early in life, the aim should be to give the greatest possible permanence to the operations, with the idea ever in mind that the highest possibilities in dental art involve saving those organs for a lifetime.

The assumption that the deciduous teeth may be neglected because they will eventually be lost should be combated at every opportunity.

There is not only the possible suffering and injury to the health resulting from diseased and abscessed teeth and lack of mastication of the food, but there is the question of acquired habits which may conduce to permanent injury. The habit of bolting the food unmasticated, and, therefore, unfit for service, often clings through life. Effective mastication is a weighty factor in the health and longevity of the individual, and it is all-important that the teeth of children should be kept in such condition as shall conduce to habits of thorough mastication. The materials for filling the deciduous teeth are limited to gutta-percha, cement, and amalgam. Cavities in the anterior teeth are usually shallow, and it is not generally possible to establish perfect outlines; the decay can only be removed, more or less thoroughly, and a material used that can be placed against the surface and remain by its own adhesive properties.

In the deciduous molars, which are retained longer, occlusal cavities are easily managed with either cement or amalgam. If cement is used it should be inserted with considerable pressure, carrying it forcibly into every groove or inequality of the surface, for which purpose the index-finger of the operator may serve. The occluso-proximal cavities are more difficult to manage because of the almost universal sensitiveness. The separation of the teeth from the expansion of the jaw and the limited contour of the fillings leads to pockets between the teeth which become a source of discomfort. In desperate cases it may be advisable to bridge across the interproximate space, for which purpose gutta-percha is excellent, though, as it is easily worn out, amalgam is probably the more serviceable material for these cavities. A great aid in securing firmness for these bridging fillings will be found in placing a metal bar across the space, building the fillings around and over it. If a pulp is exposed in a deciduous tooth, syringe well with tepid water and remove anything causing pressure on the pulp. Then apply oil of cloves on a pledget of cotton the size of a pin-head, and cover with dry cotton and fill the cavity over this. Oxide of zinc and oil of cloves makes an anodyne and antiseptic paste which may be flowed over an exposed pulp and protected by a filling of gutta-percha or cement. This will keep the pulp comfortable while it dies, and after a week or two the canals can usually be cleaned and filled. The pulps of deciduous teeth are not tenacious of life, and it does not require arsenic to kill them. If abscessed they should be cleaned by mechanical means and packed with cotton saturated with oil of cloves, and pressure made by means of unvulcanized rubber packed in the cavity till the oil of cloves comes out of the fistulous opening. The tooth may then be filled. The pulp-chamber should be flooded with a solution of gutta-percha in eucalyptol, and some temporary stopping forced into each canal until the eucalyptol shows at the opening of the fistula. They will rarely give further trouble.

The management of the permanent teeth in childhood is one of the most important problems. The teeth which suffer the most from decay are those which erupt earliest. The first permanent molars are called upon to do longer service than any other teeth, and have a very important function in the dental arch during the growth to full length of the bicuspids and second molar. If not kept in position the jaws are allowed to drop too close together, so that the upper incisors overlap the lower more than normal, and the bicuspids and second molars never acquire their full length and true position. Every effort should therefore be made to preserve the first permanent molars. On the slightest approach of caries they should be carefully filled. If they apply late, build them up, or crown if necessary, rather than yield them to the forceps. The material to be used is governed entirely by the ability of the patient or the disposition of the patient to withstand dental operations. If decay occurs on the occlusal surface during the period of the tooth's eruption through the gum the most serviceable material to check the disease is cement, which will prove effective in tiding the tooth over till it is fully erupted. Even when the flap of gum has not entirely receded, when the tendency to decay is great, it is well to use cement as a preventative, forcing it into the grooves and sulci of the occlusal surface, renewing it until conditions make it possible to insert metal fillings,—"conditions" relating to expediency and forbearance on the part of the patient to withstand tedium and pain rather than to any pronounced change of structure. As to the choice between gold and amalgam this is subject to two considerations,—one of expense, the other of ability of the patient to submit to gold operations without undue nervous strain. We must not jeopardize the nervous system of our young patients in the blind effort to live up to some high ideal. It is a question of physical and mental stamina on the part of the patient. When the area of the cavity is not too great, gold- and tin-foil rolled together is a useful material for filling these occlusal cavities, but it cannot be depended upon to wear well in cavities with a broad masticating surface.

The mesial surface of the first permanent molars calls for the most careful attention, as it is in contact with the second deciduous molar, and, if the latter is affected on its distal surface, the former is almost certain to suffer. It is well in many cases to grind away the distal surface of the deciduous tooth, rendering it more easy to keep the mesial surface of the permanent molar clean. If decay occurs it may be controlled with gutta-percha, or cement may be used, though it cannot be depended upon for any length of service. As soon as the deciduous molar is lost the permanent molar should be promptly filled with gold before the eruption of the second bicuspid, and the metallic surface should be made sufficiently broad to render the operation as permanent as possible. If the occlusal surface also is involved, a combination of gutta-percha and cement makes the best temporary filling, laying the gutta-percha over the cervical third of the cavity. The care of the permanent incisors calls for careful consideration. If decay occurs early it is usually best to resort to cement or gutta-percha, and the patient judiciously schooled towards an attitude of sufficient forbearance to submit to gold operations as early as may be practicable.

Discussion of these papers was made the special order of business at three P.M.

Dr. James Truman next read a paper entitled "Reflexes from Lower Molars."

(For Dr. Truman's paper, see Vol. XX., page 641.)

The Association then adjourned to two P. M.

(To be continued.)

